# Belladonna in Arresting Nocturnal Incontinence of Urine

**Published:** 1871-04

**Authors:** 


Belladonna in arresting Nocturnal Incontinence of Urine —The efficacy of belladonna in arresting nocturnal incontinence of urine, so troublesome an affection in children, is probably sufficiently well known. Nevertheless, the two following cases related (*Lancet*, Oct. 22, 1870) by Dr. J. B. Yeo, assistant physician to King's College Hospital, are so striking as to be well worth recording. Besides, they occurred at an age which enables us to analyze the mode of the remedy better than could be done in younger patients.

A boy, æt. 16, an out-patient at the hospital, complained of nocturnal incontinence of urine. He was a thin, delicate-looking boy, with a hectic flush on his face; and he manifested in a marked degree that *shamefacedness*, and mental distress which so embarrassing an infirmity may well produce. The accident happened every night, and had persisted ever since his infancy. He was ordered to take five minims of the tincture of belladonna, with ten minims of the tincture of the perchloride of iron, three times a day, and to avoid drinking any fluid for some hours before bedtime. After taking this prescription for a week, he reported himself as only slightly better; he had passed urine in bed three or four times since he had taken the medicine. He was ordered to take ten minims of the belladonna tincture instead of five. This reduced the frequency of the accident to first once in seven days; then once in about fourteen days; and on increasing the dose to fifteen minims, once in three weeks. With the latter dose he took no steel, as his general condition had improved marvellously. There had been, so far, little or no alteration in the dimensions of the pupils, and vision had been in no wise interfered with; but on the recurrence of a slightly increased frequency of the incontinence, he was ordered twenty minims of the tincture of belladonna three times a day. The nocturnal incontinence now completely ceased, but the vision became so indistinct that he could not "read his copy." He was therefore ordered to take only one dose of twenty minims in the twenty-four hours, and that at bedtime. He continues quite free from a return of the complaint. This patient had never suffered from any irritability of the bladder by day, and had no occasion to pass water more frequently than other people.

The next case was that of a girl, also between sixteen and seventeen years of age, who had similarly suffered ever since her infancy from nocturnal incontinence of urine. She came to King's College Hospital as an out-patient on March 5th last, and at that time she was nightly passing her urine in bed. She was living in the country, and was a fresh-looking, healthy and robust young woman; but her mental distress and embarrassment were extreme. This wretched malady unfitted her for going into service, might be justly regarded as an impediment to marriage, and altogether made her life a misery. She was put on the same kind of treatment as the preceding case, except that she at once commenced taking ten minims of the tincture of belladonna thrice daily, and she was ordered no steel, as her general contiondi was good. For the first week little or no improvement was manifested; the dose of belladonna was therefore increased to fifteen minims, which dose she has continued to take ever since. The incontinence rapidly abated, and now scarcely ever occurs. Her appearance and manner have at the same time undergone an entire change; a bright and cheerful aspect has replaced a tacituun, morose, and discontented mien. She has, for nearly a month, been engaged in a situation as a domestic servant, for which she voluntarily applied. Here then, is a remarkable instance where the whole course and prospects of a life have been rapidly changed by the administration of a few minims of a vegetable tincture!

Dr. Y. concludes that the efficacy of belladonna in these cases is owing to its influence in giving tone to the weakened sphincter vesicæ. This observation is in harmony with the statements which have recently been made as to the action of belladonna as an aperient by promoting the peristaltic contractions of the involuntary muscular fibres of the intestinal canal.—*American Jour. Med. Sciences.*

